# Iatrogenic intraprosthetic dislocation after closed reduction of dual mobility total hip arthroplasty: Report of two cases

**DOI:** 10.1016/j.ijscr.2020.04.085

**Published:** 2020-05-14

**Authors:** Marco Rotini, Marco Cianforlini, Daniele Aucone, Emanuele Pacetti, Rocco Politano

**Affiliations:** a Department of Clinical and Molecular Science, School of Medicine, Università Politecnica Delle Marche, Via Tronto, 10/A, 60126, Ancona, Italy; b Department of Orthopaedics and Traumatology, Jesi Civil Hospital, Viale Della Vittoria, 76, Jesi Ancona, Italy

**Keywords:** Total hiparthroplasty, Dual mobility, Iatrogenic, Intraprosthetic, Dislocation, Case report

## Abstract

**Introduction:**

Among hip arthroplasty, dual mobility aims to improve ROM and reduce dislocation rates, however this particular implant can fail in specific ways. Iatrogenic intraprosthetic dislocation (IPD) is a rare occurrence that can happen during closed reduction of a dislocated dual mobility total hip arthroplasty.

**Presentation of Case:**

#1 – A 34-year-old male who came to our attention with an undiagnosed IPD. He had experienced a classic dislocation 6 days earlier, which was treated with closed reduction. CT-scan confirmed decoupling of the metal head and PE liner. #2 – An 89-year-old male came to our attention for THA dislocation. During closed reduction manouvers he suffered IPD of the implant. Both patients were treated with revision surgery.

**Discussion:**

Despite being already reported in literature, IPD are still not well known to practitioners and sometimes overlooked even by orthopaedic specialists. Given the good results and diffusion of this kind of implant, iatrogenic IPD in the contest of a classic dislocation might become more frequent in the clinical practice.

**Conclusion:**

When performing reduction maneuvers for a dislocated dual mobility total hip arthroplasty, X-rays must be carefully inspected for signs of IPD which, if undiagnosed, can lead to major implant damage and the need for extensive revision surgery.

## Highlights

• Dual mobility THA is widely appreciated for its lower rate of dislocation.
• These implants can experience intraprosthetic dislocation between head and PE liner.
• Intraprosthetic dislocation can result from maneuvers during closed reduction.
• Greater attention should be paid when treating dislocated dual mobility THAs.

## 1 Introduction

Hip instability and dislocations represent the second leading cause of complication after total hip arthroplasty (THA) [1], posing a challenge for standard implants and even for the most experienced surgeons.

Dual mobility (DM) THA has been used in France since 1974 with the aim to improve ROM and reduce dislocation rate compared to fixed bearing THA. The implant is based on two principles: a small articulation (between head and PE liner) coupled with a large articulation (between PE and acetabular shell) acting as a large diameter head in order to increase jump-distance and prevent instability. The metal head is designed to be fully retained into the PE liner; however past studies demonstrated a high occurrence of separation of the two elements (intraprosthetic dislocation, IPD) due to premature wear [2,3]. In the later years, improvements such as higher resistance crosslinked PE [4] and updated neck design supposedly corrected the complications affecting early DM implants, renewing the interest for the proven lower dislocation rate of this model and encouraging a wider use [[5], [6], [7]]. Most recent studies demonstrated that with modern DM implants escape of the femoral head from the liner is a relatively rare [8,9] but meaningful event as it requires surgical treatment. Moreover, the implant failure can even be paucisymptomatic leading to metallosis and increase of metal ions serum levels [10,11] thus emphasizing the importance of a regular follow-up. However, suspect of IPD should also be raised in another setting: given its design, when a DM implant does experience a classic dislocation the reduction maneuvers can lead to iatrogenic IPD. Aim of this paper was to report 2 cases of iatrogenic IPD treated by our department (National Health Service). The work has been reported in line with the SCARE criteria [12]. This research did not receive any specific grant from funding agencies in the public, commercial, or not-for-profit sectors.

## 2 Presentation of cases

The following cases came to our attention in June 2019. Both procedures were carried out by the same surgeon, whose main field of interest and activity is hip arthroplasty.

**Fig. 1:**
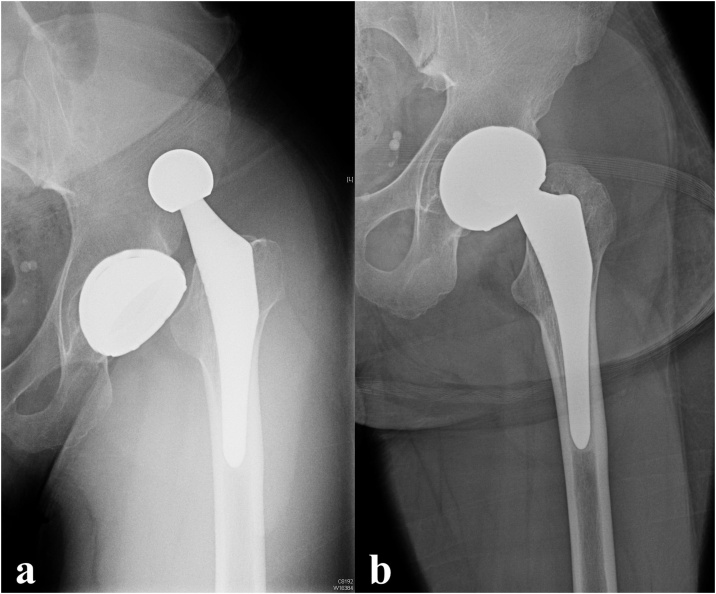
X-rays fromprevious recent hospitalization.

*Case #1:* June 2019, a 34-year-old-man presented to the emergency department of our hospital. Anamnesis: affected by Down syndrome, underwent THA with a DM implant in 2016 to address early onset osteoarthritis related to a femoral head osteonecrosis. Despite the limited collaboration, his follow-up had been regular. Six days before coming to our attention, the patient was hospitalized in a nearby institute for a first episode of hip dislocation (Fig. 1a) which was treated with closed reduction. Following clinical observation and post-reduction X-rays (Fig. 1b) the patient was discharged with the indication to continue his regular follow-up. In the following days he resumed weight bearing with mild pain, until a sudden recrudescence of symptoms to the left side which led him to our hospital.

**Fig. 2:**
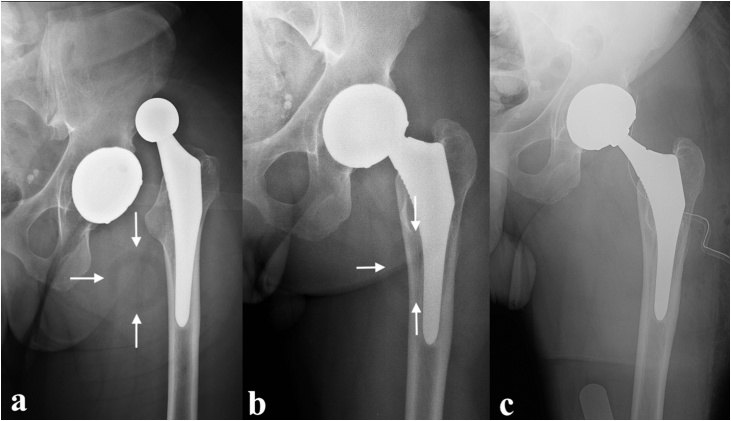
X-rays takenbefore (a) and after (b) closed reduction in our department (the arrows point to the “bubble sign”). Control radiograph after revision surgery (c).

Upon admission, radiographic evaluation showed recurrence of left THA dislocation (Fig. 2a). Closed reduction under sedation was successfully performed, however subsequent X-rays showed the implant’s head/neck was not centered in the acetabular cup and a circular radiolucent area next to the femur (also known as “bubble sign” [13]) (Fig. 2b). Both these findings were, in hindsight, also present in post-reduction X-rays from the previous hospitalization. A CT-scan was performed, which confirmed the suspect of IPD, showing direct contact of the metal head with the cup and migration of the PE liner in the anterior region of the thigh. The patient was scheduled for revision surgery. Intraoperatively, despite an area of metallosis, there was no apparent damage to the cup. The surgeon therefore proceeded to replace the femoral head with a longer neck (V40 Femoral Head 28 + 8 mm, Stryker) to obtain stability together with a new PE liner (Fig. 2c).

Case #2: June 2019, an 89-year-old-man was sent to our attention through the emergency department. Anamnesis: the patient underwent right cemented THA with DM cup in 2015 for osteoarthritis (Taperloc + Avantage, Zimmer Biomet). The patient also reported a prior femoral fracture. During the afternoon before admission, the patient experienced pain and functional impairment to the right hip while crouching.

**Fig. 3:**
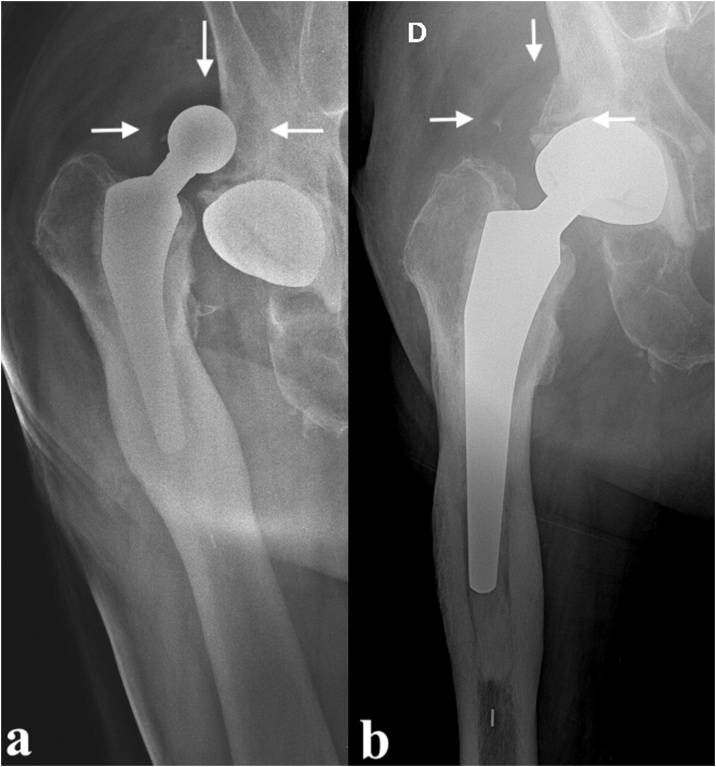
X-rays takenbefore (a) and after (b) closed reduction (the arrows point to the “bubble sign”).

**Fig. 4:**
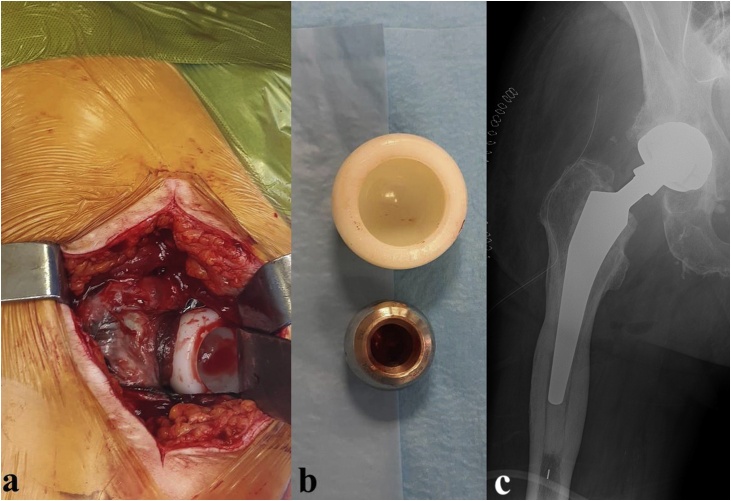
The PE liner was retrieved among soft tissues (a) with no noticeable sign of wear (b). Control radiograph after revision surgery (c).

Radiographical evaluation revealed dislocation of the implant, happening between the cup and the PE liner as the latter is clearly visible around the metal head (Fig. 3a). Closed reduction under sedation was obtained after several trials. Intra-procedural fluoroscopy was already suggestive of IPD as the femoral neck did not appear centered in the cup. Post-reduction X-rays confirmed the suspect: the PE liner lost engagement with the metal head and located just above the superior acetabular margin (Fig. 3b). Revision surgery was mandatory in this case too. The PE was retrieved showing no noticeable sign of wear (Fig. 4a–b). The acetabular cup appeared mobilized and was therefore replaced (Avantage Acetabular Cup size 50, Zimmer Biomet) together with the femoral head (BioBall 28 mm XXL, Merete) and PE liner (Fig. 4c).

## 3 Discussion

Intraprosthetic dislocations have been previously classified by Philippot et al. [14] in three types, all characterized by wear of the retentive rim linked to different causes: type 1 is a detachment of head from the liner due to pure wear with no signs of arthrofibrosis or cup loosening, type 2 is induced by a blockage of the liner due to extrinsic tissue (usually arthrofibrosis or ossifications), and type 3 is associated with cup loosening. This classification accounts for spontaneous IPDs but does not consider iatrogenic IPDs which might happen during surgery due to poor assembly of components or during closed reduction of a dislocated DM bearing when the PE liner locks on the metal cup or any other pelvic prominence, leading to the same “bottle-opener” effect described for hemiarthroplasty. At the present time, wear related late IPDs are becoming increasingly rare thanks to design and material advances [9]. This was examined in a recent systematic review which found no case of IPD in primary DM THAs performed after 2007 [15]; similar results were obtained from other recent studies [[16], [17], [18]]. On the other hand, with the decrease of wear related dislocations, iatrogenic ones now account for the majority of IPDs and might represent the main failure mode of these devices going forward [19,20]. For this reason, it’s important for practitioners to identify dual mobility implants, understand the risk for iatrogenic IPD, prepare for a more cautious closed reduction and be able to identify the case when it occurs. The reported “bubble sign”, although highly suggestive of IPD, can sometimes be absent of challenging to identify on plain X-rays when overlapping with bony structures. Indeed, while this sign is clearly visible on X-rays of other reports, in our cases it was subtle and easily missable. Therefore, any closed reduction suspicious for IPD should be investigated with CT-scan to confirm or exclude the case. Accurate diagnosis of IPD is even more important when we account for the fact that this situation is not always frankly symptomatic, with patients reporting simple discomfort and weakness of the leg [10]. When combined with the fact that absence of PE liner between metal head and cup can lead to metallosis and damage to the cup, converting a component exchange surgery into a more complex revision surgery, the importance of prompt diagnosis appears clear.

Dual mobility bearings, thanks to their size, are more stable but also prone to get entangled and suffer the “bottle-opener” effect. Theoretically, inaccurate assembly of the PE liner and metal head during primary surgery might later influence the likeness of an IPD during closed reduction of a dislocation. In such a case the liner would have less retentive action, therefore extreme attention must be paid to components assembly in DM implants. Finally, the best strategy to approach a dislocated DM implant is yet to be determined. Some authors suggest using a deeper anesthesia such as general or spinal anesthesia, which ensure complete muscle relaxation. This could allow for gentler manipulation, reducing the energy involved in reduction maneuvers and therefore likeness of IPD [19,20].

## 4 Conclusions

With this case report we wanted to raise awareness on a relatively rare but significant complication specific of DM implants. Given the good results and diffusion of this kind of implant, iatrogenic IPD in the contest of a classic dislocation might become more frequent in the clinical practice. Recent literature suggests special caution and general anesthesia should be employed during closed reduction. After maneuvers, X-rays must be carefully inspected for signs of IPD which if undiagnosed can lead to major implant damage and the need for extensive revision surgery.

## Funding

None.

## CRediT authorship contribution statement

**Marco Rotini:** Data curation, Writing - original draft, Visualization. **Marco Cianforlini:** Supervision, Writing - review & editing. **Daniele Aucone:** Investigation, Resources. **Emanuele Pacetti:** Investigation, Resources. **Rocco Politano:** Conceptualization, Validation.

## Declaration of Competing Interest

None.
